# Supplementary material for: Identification of photoperiod-induced specific miRNAs in the adrenal glands of Sunite sheep (Ovis aries)
Source: Front Vet Sci. 2022 Jul 22;9:888207. doi: 10.3389/fvets.2022.888207 (PMC9354845; doi:10.3389/fvets.2022.888207)
Supplement: Supplementary file 1 [file Data_Sheet_1.ZIP › Supplementary file/miRDeep2 file.zip!/miRDeep2 file/Know_Structure/SP42A3/oar-mir-665.pdf]

miRBase precursor : oar-mir-665
Total read count : 49
oar-miR-665-5p read count : 2
oar-miR-665-3p read count : 46
remaining reads : 1

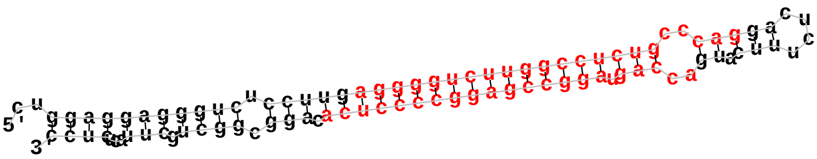

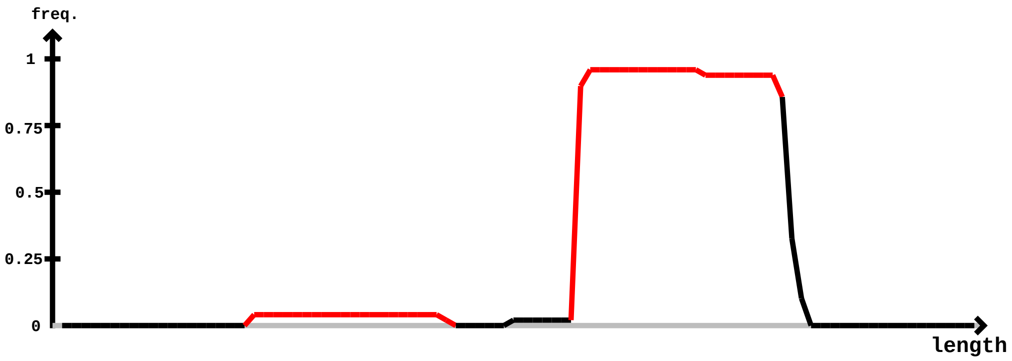


```
                                                      oar-miR-665-3p
                    oar-miR-665-5p
5'- cuggaggagggucuccuugaggggucuuggccucugcccaggacucuuucaugaccaguaggccgaggccccucacaggcggcugcuuacucucc   -3'    exp
    ..((((((((((.(((((((((((((((((((((((..(((((....))).))..))).)))))))))))))))).))).)))).))).))))..    reads   mm      sample
    ...................aggggucuuggccucugccc........................................................    1       0       seq
    ...................aggggucuuggccucugccca.......................................................    1       0       seq
    ..............................................uuucaugaccaguaggccga.............................    1       0       seq
    .....................................................accaguaggccgaggccccuc.....................    4       0       seq
    .....................................................accaguaggccgaggccccuca....................    25      0       seq
    .....................................................accaguaggccgaggccccucac...................    9       0       seq
    .....................................................accaguaggccgaggccccucaca..................    5       0       seq
    ......................................................ccaguaggccgaggccccuca....................    1       0       seq
    ......................................................ccaguaggccgaggccccucac...................    2       0       seq
```
